# The association between systemic inflammatory response index and new-onset atrial fibrillation in patients with ST-elevated myocardial infarction treated with percutaneous coronary intervention

**DOI:** 10.1186/s12872-022-02989-9

**Published:** 2022-12-06

**Authors:** Jingfeng Wang, Sisi Hu, Cheng Liang, Yang Ling

**Affiliations:** Department of Cardiology, Yijishan Hospital Affiliated to Wannan Medical College, 2# West Zhe Shan Road, Wuhu, 241000 China

**Keywords:** Systemic inflammatory response index, New-onset atrial fibrillation, ST-elevated myocardial infarction, Percutaneous coronary intervention, Prognosis

## Abstract

**Background:**

New-onset atrial fibrillation (NOAF) complicating with ST-elevated myocardial infarction (STEMI) patients following percutaneous coronary intervention (PCI) is associated with worse prognosis. The systemic inflammatory response index (SIRI), serves as a novel inflammatory indicator, is found to be predictive of adverse outcomes. The aim of this study is to explore the association between NOAF and SIRI.

**Methods:**

A retrospective data included 616 STEMI participants treated with PCI in our cardiology department had been analyzed in present investigation, of which being divided into a NOAF or sinus rhythm (SR) group based on the presence or absence of atrial fibrillation. The predictive role of SIRI for in detecting NOAF had been evaluated by the logistic regression analyses and receiver operating characteristic (ROC) curve. Additionally, long-term all-cause mortality between both groups was compared using the Kaplan–Meier test.

**Results:**

NOAF during hospitalization developed in 7.6% of PCI-treated individuals. After multivariate regression analyses, SIRI remains to be an independently predictor of NOAF (odds ratio 1.782, 95% confidence interval 1.675–1.906, *P* = 0.001). In the ROC curve analysis, SIRI with a cut-off value of 4.86 was calculated to predict NOAF, with 4.86, with a sensitivity of 80.85% and a specificity of 75.57%, respectively (area under the curve (AUC) = 0.826, *P* < 0.001). Furthermore, pairwise compassion of ROC curves displayed the superiority of SIRI in the prediction of NOAF in comparison with that of neutrophil/lymphocyte or monocyte/lymphocyte (*P* < 0.05). In addition, the participants in NOAF group had a significantly higher incidence of all-cause death compared to those in SR group after a median of 40-month follow-up (22.0% vs 5.8%, log-rank *P* < 0.001).

**Conclusion:**

SIRI can independently predict NOAF in patients with STEMI after PCI, with being positively correlated to worsened outcomes.

## Background

New-onset atrial fibrillation (NOAF) is one of the most common arrhythmic complications in the setting of ST-elevated myocardial infarction (STEMI) following percutaneous coronary intervention (PCI), with a reported incidence ranging from 3.0 to 13.7% [1–3]. Prior clinical trials demonstrated that NOAF is significantly associated with worse short- and long-term prognosis in STEMI patients during the era of PCI [1, 3–6]. Thus, it is of utmost importance to effectively assess and predict the risk of NOAF in STEMI patients following PCI.

Inflammatory response and immune system cells, such as monocyte, neutrophil and lymphocyte, have been implicated in the initiation and development of atrial fibrillation (AF) [7–10]. Furthermore, the main ratios, including neutrophil-lymphocyte ration (NLR) and lymphocyte-monocyte ration (MLR) have been shown to be independently predictive of AF [11–13]. Recently, the systemic inflammatory response index (SIRI), deprived from three peripheral blood inflammatory cells, namely, neutrophil, lymphocyte, monocyte, has been used to predict prognosis in both cardiovascular and non-cardiovascular diseases [14–17]. Besides that, a recent investigation shown that SIRI is positively associated with the occurrence of AF among the stroke patients [18]. However, whether SIRI is an independent risk factor of NOAF in STEMI patients remains unknown. Herein, the aim of this investigation is to explore the association between SIRI and NOAF in patients with STEMI after PCI.

## Methods

### Study population

**Fig. 1:**
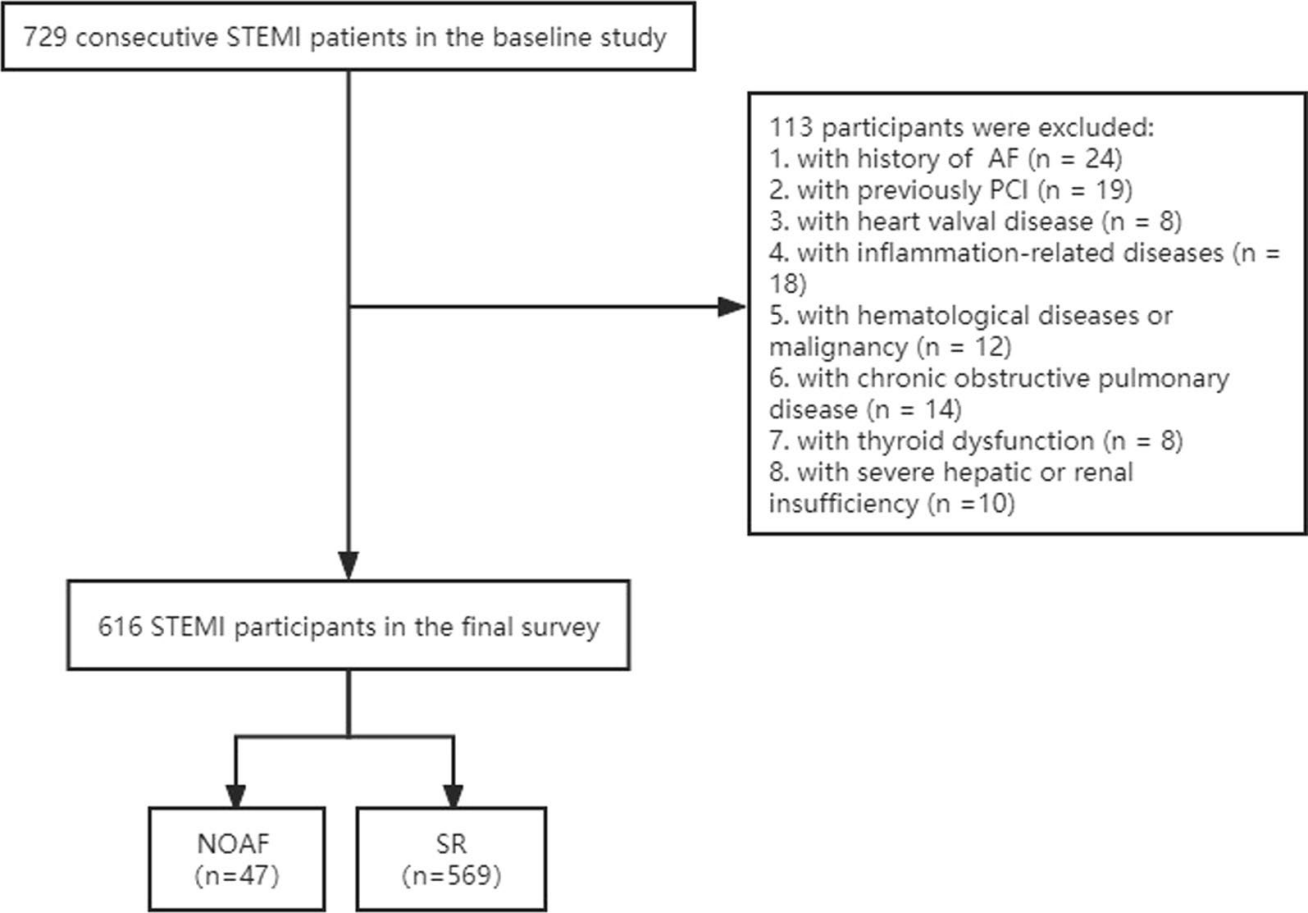
Flow chart of the population. STEMI, ST-elevated myocardial infarction; PCI, Percutaneous coronary intervention; AF, Atrial fibrillation; NOAF, New-onset atrial fibrillation; SR, Sinus rhythm

A total of consecutive STEMI participants admitted to the Department of Cardiology between February 2016 and December 2021 had been recruited in this retrospective study. All STEMI patients aged over 18 years were treated invasively within the first 24 h after symptoms onset. Exclusion criteria contained individuals with previously paroxysmal/non-paroxysmal AF, history of PCI, heart valve disease, inflammatory-related diseases, hematological diseases, malignancy, chronic obstructive pulmonary disease, hyperthyroidism or hypothyroidism, and severe hepatic or renal dysfunction. Finally, the study cohort was comprised of 616 eligible participants (Fig. 1). This study was approved by the Institutional Review Board of Yijishan Hospital Affiliated of Wannan Medical College and complied with the principles set forth by the Declaration of Helsinki. The written informed consent was waived due to the nature of retrospective study design.

### Demographic, laboratorial, echocardiographic data

Demographic variables, including age, gender, height, weight, a history of diabetes mellitus (DM) or hypertension, drinking or smoking status, were obtained from a questionnaire by trained doctors. Routine blood analyses, such as, the levels of neutrophil counts, lymphocyte counts, monocyte counts, were performed before PCI and measured with an automated blood count analyzer (Mindray BC-5380, Shenzhen, China). Biochemical markers, including glucose, triglyceride, total cholesterol, high-density lipoprotein cholesterol (HDL-c), low-density lipoprotein cholesterol (LDL-c), creatinine, creatine kinase (CK), and albumin were additionally analyzed for all patients after fasting for overnight by using an automated chemistry analyzer (Roche cobas E601, Basel, Switzerland). The value of NLR and MLR was defined as the counts of neutrophil and monocyte to the counts of lymphocyte, respectively. SIRI was calculated as follows, (N × M)/L, where N, M, and L was presented as the counts of neutrophil, monocyte, and lymphocyte, respectively [17]. Body mass index (BMI) was defined as body weight divided by the square of body height. The Modification of Diet in Renal Disease (MDRD) equation was calculated for all patients to evaluate the estimated glomerular filtration rate (eGFR) [19]. Transthoracic echocardiography was used to assess the diameter of left atrium (LA) and left ventricular ejection fraction (LVEF) for all patients within the first 24 h of hospitalization.

### Angiographic analyses and post-PCI management

The treatment of PCI was performed for all STEMI patients according to the contemporary guidelines [20]. The degree of coronary stenosis was quantitatively determined by synergy between PCI with taxus and cardiac surgery (SYNTAX) score [21], while the flow grades were evaluated based on thrombolysis in myocardial infarction (TIMI) criteria. All procedures of PCI were analyzed by two experienced cardiologists who were blind to the patients’ data. All STEMI patients following PCI were orally administrated with anti-platelet, statin, beta-blocker, angiotensin-converting enzyme inhibitor/angiotensin receptor blocker (ACEI/ARB), and mineralocorticoid receptor antagonist (MRA) without contraindications [20].

### Arrhythmic outcomes

All STEMI patients treated with PCI were monitored by telemetry electrocardiogram (ECG) during hospitalization at the coronary unit. Subsequently, any suspected symptom of arrhythmia was assessed by a 12-lead ECG at the ward. AF was defined as irregular RR intervals with the absence of discrete P waves, complicating by fibrillatory waves. NOAF was identified as the occurrence of an AF episode lasting 30 s during hospital period. A total of 40 NOAF patients had been treated with either electrical or chemical cardioversion, of note, amiodarone intravenously is regarded as the pharmacologic treatment of AF. Ventricular tachycardia (VT) was presented as any VT event lasting ≥ 16 consecutive beats with a rate more than 125 beat/min [22].

### Clinical endpoints

The primary adverse outcomes during hospitalization were comprised of cerebrovascular events, pulmonary edema, cardiogenic shock, VT, mortality. The long-term endpoint with respect to all-cause death was collected from medical records or obtained by telephonic interviews. Participants were followed-up from the enrollment to June 15, 2022.

### Statistical analysis

Kolmogorov–Smirnov test was performed to evaluate the distribution patterns of the continuous variables. Continuous data were defined as mean ± standard deviation or median (interquartile range) based on whether variables are normally distributed or not, whereas the categorical variables were expressed as numbers (percentages). The comparison of parametric variables was conducted by the Student’s t or Mann–Whitney U-test, as appropriate, while non-parametric data was compared using the Chi-squared or Fisher’s exact test. Multivariable logistic regression analyses, which incorporated potential risk factors originated from univariable regression analysis (all *P* < 0.05), were used to determine the independent predictors of NOAF. Receiver operating characteristic (ROC) curve and area under the curve (AUC) were generated to define the predictability of potential factors. The comparison of ROC curves for different risk factors was conducted by the DeLong method. Kaplan–Meier curves were performed to estimate time-to-event data, while the comparison of different groups was evaluated using a log-rank test. All data were analyzed by IBM SPSS 23.0, with *P* < 0.05 of statistical significance.

## Results

A total of consecutive 616 patients with STEMI underwent PCI had been recruited in this study, of whom 47 presented with NOAF. The baseline characteristics of the study population with and without NOAF are listed in Table 1. STEMI patients who developed NOAF were more prone to older, presented a higher level of BMI, had a higher prevalence of DM, and experienced a greater incidence of Killip class ≥ 2 in comparison to those with SR. With regards to biomarkers, the values of white blood cell counts, neutrophil counts, monocyte counts, NLR, MLR, SIRI, glucose, and HDL-c were significantly higher in individuals who suffered from NOAF compared with NOAF-free subjects, while NOAF patients had significantly lower levels of hemoglobin, lymphocyte counts, platelet counts, LDL-c, eGFR as well as albumin compared to non-AF patients. Additionally, dilated left atrium and weaken left ventricular ejection fraction were more prevalent in NOAF group versus SR group. As for medicine intake during hospitalization, patients with NOAF were less likely to receive beta-blocker and more likely to take MRA in comparison to SR patients. Importantly, NOAF cases tend to experience a higher probability of in-hospital adverse outcomes, including, cerebrovascular events, pulmonary edema, cardiogenic shock, ventricular tachycardia and mortality compared with patients in SR group. No significant differences were observed between the two groups in respect to gender, history of hypertension, smoking and alcohol consumption, the levels of total cholesterol, triglyceride, as well as peak creatine kinase, culprit vessels, TIMI flow pre-PCI, the values of SYNTAX score and residual SYNTAX score, the use of ACEI or ARB, and statin.

**Table 1:** Baseline characteristics of study population based on the absence or presence of NOAF

	SR (n = 569)	NOAF (n = 47)	*P*
Age (years)	63 (52–72)	71 (66–77)	< 0.001
Male, n (%)	462 (81.2)	33 (70.2)	0.069
BMI (kg/m^2^)	24.8 (23.7–26.3)	25.4 (24.7–27.1)	0.038
Hypertension, n (%)	278 (48.9)	29 (61.7)	0.091
Diabetes mellitus, n (%)	100 (17.6)	16 (34.0)	0.006
Smoking, n (%)	305 (53.6)	21 (44.7)	0.239
Alcohol consumption, n (%)	165 (29.0)	16 (34.0)	0.573
Killip class ≥ 2, n (%)	95 (16.7)	23 (48.9)	< 0.001
White blood cell (10^9^/l)	10.80 (8.80–12.85)	13.30 (10.50–16.10)	< 0.001
Hemoglobin (g/l)	143.0 (129.0–155.0)	133.0 (122.0–142.0)	0.003
Lymphocyte (10^9^/l)	1.2 (0.9–1.7)	0.8 (0.3–1.2)	< 0.001
Neutrophil (10^9^/l)	8.70 (6.70–10.80)	11.20 (8.30–13.70)	< 0.001
Monocyte (10^9^/l)	0.40 (0.30–0.60)	0.70 (0.40–1.00)	< 0.001
Platelet (10^9^/l)	178.00(142.50–222.50)	153.00(122.00–196.00)	0.019
NLR	7.58 (4.53–11.41)	14.38 (7.56–38.00)	< 0.001
MLR	0.33 (0.24–0.49)	0.78 (0.50–2.75)	< 0.001
SIRI	2.80 (1.69–4.80)	7.38 (5.11–24.00)	< 0.001
Glucose (mmol/l)	5.57 (4.79–6.87)	7.38 (5.57–9.62)	< 0.001
Total cholesterol (mmol/l)	4.13 (3.55–4.89)	3.89 (3.20–4.53)	0.068
Triglyceride (mmol/l)	1.46 (1.04–2.08)	1.63 (1.22–2.08)	0.282
HDL-c (mmol/l)	1.16 (1.02–1.32)	1.31 (1.15–1.44)	0.002
LDL-c (mmol/l)	2.40 (2.01–2.96)	2.06 (1.70–2.63)	0.005
eGFR (ml/min*1.73m^2^)	121.78 (97.69–149.71)	94.82 (83.22–130.24)	0.001
Peak CK (10^3^U/L)	1.59 (0.85–2.76)	1.84 (0.99–3.46)	0.244
Albumin (g/l)	36.48 ± 3.69	33.86 ± 3.15	< 0.001
Culprit vessels, n (%)			
LAD	310 (54.5)	24 (51.1)	0.764
LCX	48 (8.4)	2 (4.3)	0.465
RCA	211 (37.1)	21 (44.7)	0.301
TIMI 3 flow pre-PCI, n (%)	117 (20.6)	6 (12.8)	0.199
Stent-length (mm)	29 (21–33)	29 (23–36)	0.055
SYNTAX score	19.0 (13.0–23.5)	21.0 (15.0–25.5)	0.050
Residual SYNTAX score	4.0 (0–9.0)	5.0 (2.0–12.0)	0.070
LA (mm)	36 (34–40)	40 (38–42)	< 0.001
LVEF (%)	52 (48–56)	48 (42–55)	0.002
Medications in-hospital			
ACEI/ARB, n (%)	276 (48.7)	24 (52.2)	0.762
Beta-blocker, n (%)	372 (65.6)	20 (43.5)	0.003
MRA, n (%)	98 (17.3)	19 (41.3)	< 0.001
Statin, n (%)	515 (90.5)	42 (89.4)	0.797
In-hospital outcomes			
Cerebrovascular events, n (%)	5 (0.9)	2 (4.3)	0.036
Pulmonary edema, n (%)	55 (9.7)	20 (42.6)	< 0.001
Cardiogenic shock, n (%)	62 (10.9)	15 (31.9)	< 0.001
Mortality, n (%)	17 (3.0)	6 (12.8)	0.001
VT, n (%)	16 (2.9)	6 (13.0)	< 0.001

NOAF, New-onset atrial fibrillation; SR, Sinus rhythm; BMI, Body mass index; NLR, Neutrophil/lymphocyte ratio; MLR, Monocyte/lymphocyte ratio; SIRI, System inflammation response index; eGFR, Estimated glomerular filtration rate; HDL-c, High-density lipoprotein cholesterol; LDL-c, Low-density lipoprotein cholesterol; CK, Creatine kinase; LAD, Left anterior descending coronary artery; LCX, Left circumflex coronary artery; RCA, Right coronary artery; TIMI, Thrombolysis in myocardial infarction; SYNTAX, SYNergy between percutaneous coronary intervention with TAXus and cardiac surgery; LA, Left atrium; LVEF, Left ventricular ejection fraction; ACEI, Angiotensin-converting enzyme inhibitor; ARB, Angiotensin receptor blocker; MRA, Mineralocorticoid receptor antagonist; PCI, Percutaneous coronary intervention; VT, Ventricular tachycardia

**Fig. 2:**
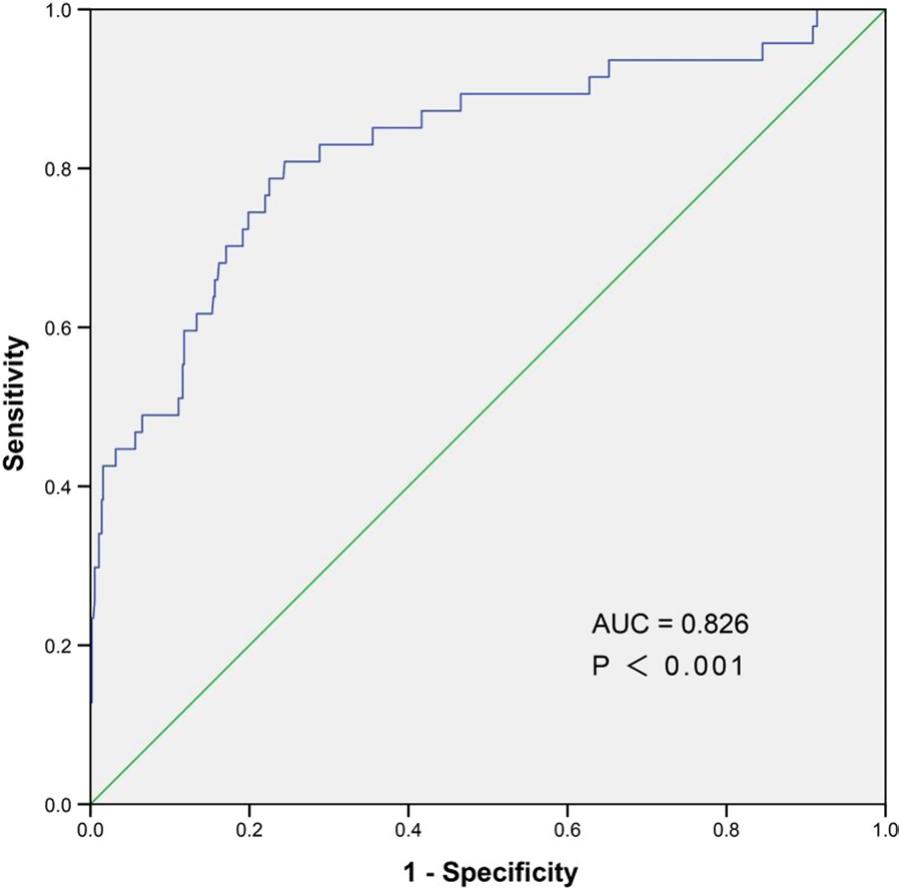
Receiver operating characteristic curve analysis demonstrated the optimal cut-off value of SIRI in predicting NOAF was 4.86, with a sensitivity of 80.85% and a specificity of 75.57%, respectively (area under the curve (AUC) = 0.826, 95% confidence interval 0.755–0.898, *P* < 0.001). SIRI, Systemic inflammatory response index; NOAF, New-onset atrial fibrillation

**Fig. 3:**
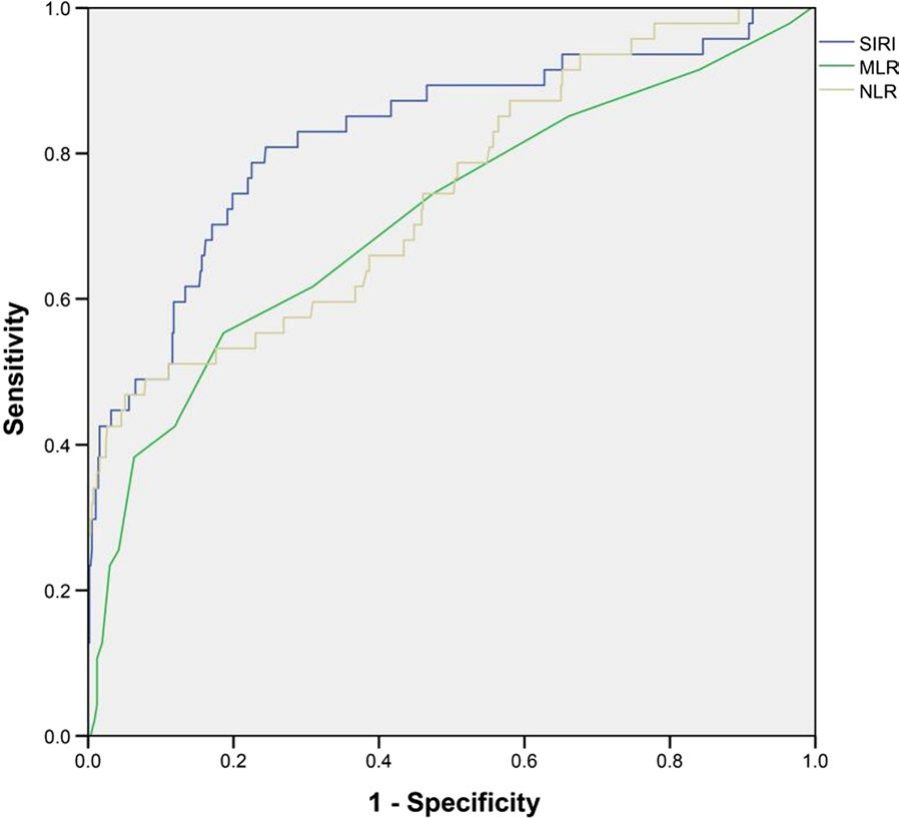
Receiver operating characteristic curves with area under the curves for SIRI, NLR, MLR to predict NOAF in STEMI patients treated with PCI, indicating that the prognostic role of SIRI in identifying NOAF is positively superior to that of NLR or MLR, respectively (*P* < 0.05). SIRI, Systemic inflammatory response index; NLR, Neutrophil/lymphocyte; MLR, Monocyte/lymphocyte; NOAF, New-onset atrial fibrillation; STEMI, ST-elevated myocardial infarction; PCI, Percutaneous coronary intervention

In univariate logistic regression analysis, several risk factors, including SIRI, age, Killip class ≥ 2, DM, cardiogenic shock, white blood cell, hemoglobin, lymphocyte, neutrophil, monocyte, NLR, MLR, albumin, glucose, HDL-c, LDL-c, eGFR, stent-length, SYNTAX score, residual SYNTAX score, left atrium, left ventricular ejection fraction and the use of beta-blocker as well as MRA, had been identified as significant predictors of NOAF (all *P* values < 0.05). Further multivariate analyses demonstrated that the values of SIRI, albumin, glucose and eGFR were the independent predictors of NOAF in STEMI treated with PCI (Table 2). Receiver operator characteristics curve analysis displayed that the cut-off value of SIRI for the prediction of NOAF in STEMI patients underwent PCI was 4.86, with a sensitivity of 80.85% and a specificity of 75.57% (AUC = 0.826, *P* < 0.001, as shown in Fig. 2). The DeLong test displayed that the predictive role of SIRI was significantly superior to that of NLR or MLR (AUC_SIRI_ vs AUC_NLR_ Z test = 2.900, *P* < 0.05; AUC_SIRI_ vs AUC_MLR_ Z test = 2.763, *P* < 0.05; Fig. 3).

**Table 2:** Univariate and multivariate analysis and predictors of NOAF variable univariate analysis multivariate analysis

	OR	95% CI	*P* value	OR	95% CI	*P* value
SIRI	1.160	1.103–1.221	< 0.001	1.782	1.675–1.906	0.001
Age, years	1.072	1.041–1.103	< 0.001			
Male	0.546	0.282–1.056	0.072			
BMI, kg/m^2^	1.171	0.999–1.373	0.052			
Killip class ≥ 2	4.782	2.591–8.826	< 0.001			
Hypertension	1.686	0.916–3.106	0.093			
Diabetes mellitus	2.421	1.275–4.594	0.007			
Smoking	0.699	0.384–1.272	0.241			
Alcohol consumption	1.264	0.673–2.373	0.466			
Cardiogenic shock	3.833	1.966–7.473	< 0.001			
Biochemical indicators						
White blood cells, 10^9^/L	1.165	1.085–1.251	< 0.001			
Hemoglobin (g/l)	0.977	0.959–0.995	0.011			
Lymphocyte (10^9^/l)	0.190	0.094–0.382	< 0.001			
Neutrophil (10^9^/l)	1.120	1.048–1.197	0.001			
Monocyte (10^9^/l)	11.453	4.650-28.209	< 0.001			
Platelet, 10^9^/L	0.997	0.992–1.003	0.308			
NLR	1.125	1.080–1.171	< 0.001			
MLR	10.320	4.705–22.635	< 0.001			
Albumin, g/L	0.818	0.749–0.892	< 0.001	0.820	0.715–0.940	0.004
Glucose, mmol/L	1.146	1.070–1.228	< 0.001	1.129	1.022–1.246	0.017
Total cholesterol, mmol/L	0.768	0.586–1.006	0.056			
Triglyceride, mmol/L	1.018	0.795–1.304	0.885			
HDL-c, mmol/L	4.492	1.574–12.815	0.005			
LDL-c, mmol/L	0.554	0.360–0.851	0.007			
eGFR, (ml/min*1.73m^2^)	0.988	0.979–0.996	0.005	0.985	0.972–0.997	0.015
Peak CK (*10^3^U/L)	1.000	1.000–1.000	0.149			
Coronary angiography						
Culprit vessel LAD^a^	0.778	0.422–1.433	0.421			
Culprit vessel LCX^a^	0.419	0.095–1.846	0.250			
TIMI 3 flow pre-PCI	1.769	0.733–4.267	0.204			
Stent-length	1.022	1.004–1.040	0.018			
SYNTAX score	1.055	1.012-1.100	0.012			
Residual SYNTAX score	1.054	1.005–1.105	0.03			
LA	1.132	1.063–1.206	< 0.001			
LVEF	0.938	0.902–0.975	0.001			
Medication during hospitalization						
ACEI/ARB	1.150	0.630–2.099	0.648			
Beta-blockers	0.403	0.219–0.741	0.003			
MRA	3.368	1.801–6.298	< 0.001			
Statin	0.881	0.334–2.320	0.797			

NOAF, New-onset atrial fibrillation; SIRI, System inflammation response index; BMI, Body mass index; NLR, Neutrophil/lymphocyte ratio; MLR, Monocyte/lymphocyte ratio; HDL-c, High-density lipoprotein cholesterol; LDL-c, Low-density lipoprotein cholesterol; eGFR, Estimated glomerular filtration rate; CK, Creatine kinase; LAD, Left anterior descending coronary artery; LCX, Left circumflex coronary artery; RCA, Right coronary artery; TIMI, Thrombolysis in myocardial infarction; SYNTAX, SYNergy between percutaneous coronary intervention with TAXus and cardiac surgery; LA, Left atrium; LVEF, Left ventricular ejection fraction; ACEI, Angiotensin-converting enzyme inhibitor; ARB, Angiotensin receptor blocker; MRA, Mineralocorticoid receptor antagonist; PCI, Percutaneous coronary intervention

^a^Compared with culprit vessel RCA

**Fig. 4:**
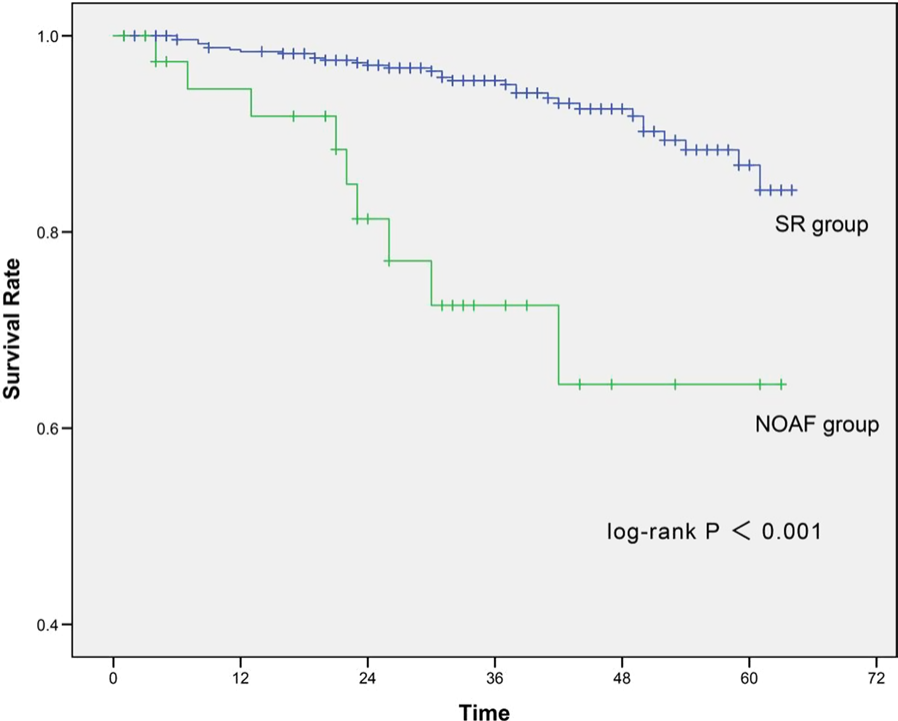
The Kaplan–Meier curves of long-term all-cause mortality between NOAF and SR group (22.0% versus 5.8%, log-rank *P* < 0.001). NOAF, New-onset atrial fibrillation; SR, Sinus rhythm

During the median period of 40-month follow-up, 41 patients (6.9%) experienced all-cause death after hospital discharge. Kaplan–Meier analysis showed that the all-cause mortality in NOAF group is significantly higher than that in SR group (22.0% versus 5.8%, log-rank *P* < 0.001, Fig. 4).

## Discussion

The aim of the present study is to explore the association between SIRI and NOAF in STEMI patients treated with PCI. Our results clearly demonstrate that the value of SIRI in NOAF group is significantly higher than that in SR group. Meanwhile, SIRI is found to be independently predictive of NOAF. Additionally, other predictors, including, albumin, glucose, and eGFR, have been found to be significantly associated with NOAF in STEMI patients following PCI. Furthermore, those with NOAF tend to experience a higher incidence of in-hospital complications and long-term all-cause mortality in comparison to those with SR.

Prior studies had proposed that NOAF occurs in a sizeable proportion of STEMI patients following PCI, ranging from 3.0 to 13.7% [1–3]. Furthermore, several researches also had demonstrated that NOAF complicating STEMI patients treated with PCI is significantly associated with worse prognosis during follow-up. An investigation of 3281 individuals in the HORIZONS-AMI study had shown that NOAF in STEMI patients following PCI is significantly correlated with higher incidences of mortality, reinfarction, target vessel revascularization for ischemia, stroke, and major bleeding during a 3-year follow-up period [6]. Madsen et al. further revealed that NOAF was closely associated with an increased risk of long-term mortality, bleeding, ischemic stroke in STEMI participants after PCI with a median follow-up of 5.8 years [5]. Besides that, Mrdovic et al. analyzed a longitudinal, single-center survey of 2096 patients over a 30-day follow-up period and discovered NOAF to be related to a higher risk of major adverse cardiovascular events [4]. Another multicenter study enrolled 786 STEMI patients presented NOAF to be an independent predictor of a composite of all-cause mortality, nonfatal reinfarction, and congestive heart failure within 1 year [23]. Similarly, our results had found that the incidence of NOAF in STEMI patients after PCI during hospitalization is approximately 7.6%, which is in line with the previously reported rates. Moreover, we also concluded that the STEMI patients in NOAF group are significantly correlated to increased risks of in-hospital complications and long-term all-cause mortality. Therefore, it is of necessity for clinicians to effectively predict and evaluate the risk of NOAF in STEMI patients underwent PCI.

Undoubtedly, inflammation plays a vital role in the initiation and development of STEMI, and also is a significant promoter for adverse cardiovascular outcomes in STEMI patients. Several inflammatory indexes, such as, C-reactive protein, NLR, interleukins, have been demonstrated to be predictive of short- or long-term prognosis in patients with STEMI [24–28]. Recent studies had demonstrated the positive relationship between SIRI and prognosis information in different subject cohorts [14–16, 18, 29]. For instance, Xu et al. conducted a multi-center retrospective analysis of 1,499 rheumatoid arthritis (RA) patients, and determined that SIRI is significantly related to the disease activity of RA, even can predict the tumor development of RA individuals [29]. Another retrospective study of 574 cases with pancreatic cancer from the Fudan University Shanghai Cancer Center found SIRI to be predictive of survival outcomes in patients with pancreatic adenocarcinomas who receive chemotherapy [15]. Zhang et al. further revealed that increased SIRI was closely related to higher risk of mortality, sepsis, and stroke severity in 2,450 stroke participants after adjusting potential confounding variables [14]. Recently, Jin et al. analyzed a cohort survey of 85,154 individuals over a 10-year follow-up period and demonstrated the positive association between SIRI and cardiovascular outcomes as well as all-cause death [17]. Han et al. analyzed 1,724 patients exhibiting acute coronary syndrome over a median follow-up of 927 days, and displayed SIRI to be independently predictive of major adverse cardiovascular events [16]. Another recent retrospective study recruited 526 individuals diagnosed as ischemic stroke proposed that SIRI is an independent predictor of AF [18]. However, the association between SIRI and NOAF in STEMI patients after PCI remains unexplored. Herein, our results had shown that the values of SIRI in NOAF group are significantly higher than those in SR group. Furthermore, we had demonstrated that elevated SIRI can independently predict NOAF in STEMI patients treated with PCI.

The specific mechanism of NOAF in STEMI populations after PCI is still uncertain. Recently prior studies had proposed that inflammation exerts a vital role in the initiation and development of AF in AMI patients [7, 8, 10]. Several inflammatory biomarkers including C-reactive protein, NLR, MHR, were found to be significantly correlated with the occurrence of AF in AMI patients [7, 12], however, the potential inflammatory indicators had been incompletely elucidated. The systemic immune-inflammation index (SII), had been found to be independently predictive of contrast-induced nephropathy in STEMI patients following PCI [30]. Additionally, a retrospective analysis of 402 STEMI individuals treated with PCI had demonstrated that SII can independently predict NOAF [31]. A retrospective and cross-sectional investigation of 1484 STEMI individuals following PCI conducted by Selçuk et al. had displayed that the value of uric acid/albumin can independently predict the development of NOAF [32]. Another recent investigation concluded that SII is an independent predictor of postoperative AF after analyzing 391 consecutive cases treated with isolated coronary artery bypass grafting and also demonstrated that the predictive role of SII is superior to that of NLR or platelet to lymphocyte ratio[33]. Yesin et al. analyzed the retrospective data which recruited 1153 STEMI individuals following PCI and demonstrated that C-reactive protein to albumin ratio can independently predict NOAF after adjusting for potential factors [34]. Recently, SIRI, which is a composite indicator that includes three inflammatory cells, namely, neutrophil, lymphocyte, and monocyte, is a convenient, inexpensive and easy biomarker in the assessment of inflammatory status. Besides that, SIRI is comprised of NLR and MLR, indicating that the predictive role of SIRI might be more comprehensive and sensitive than any of these alone. SIRI is thus an easily accessible indicator of inflammation that is proper for routine clinical application. In the present study, ROC analysis indicated SIRI present the highest AUC levels, meaning that it had better discriminative power in comparison to NLR, NLR. Furthermore, SIRI is found to be independently predictive of NOAF after adjusting for NLR, MLR and other potential risk factors.

The exact theorical base for the relation between SIRI and NOAF remains to be fully explored. Given that SIRI incorporates three immune cells, this may explain its underlying relation to NOAF in STEMI population. Monocyte accumulation serves as the source of several inflammatory cytokines, including interleukin (IL)-6, IL-1β, tumor necrosis factor (TNF)-α, thus leading to both structure and electrical atrial remodeling, which can provide the occurrence of AF with necessary substance [35–37]. Additionally, increased neutrophils located in the myocardial interstitium are bale to secrete proinflammatory cytokines, such as, proteases, peroxidases, reactive oxygen species, which induce the differentiation of fibroblasts and the production of extracellular matrix proteins, resulting in atrial fibrosis [9, 38]. The presence of lymphopenia in the context of AMI is secondary to the increased production of cortisol and elevated degree of lymphocyte apoptosis [39, 40], further resulting in the secretion of proinflammatory indicators including TNF-αand IL-6 [41]. Despite the foregoing reasonable explanation regarding the correlation between inflammation and NOAF, however, further investigation will be needed to illustrate how SIRI is associated with the development of AF.

Several clinical implications have been stated according to the present results. First, SRI is a relatively common and easily obtained biomarker in clinical practice. Further, it might be beneficial for clinicians to efficiently identify the patients with high risk of NOAF by using the pre-procedural SIRI, thus providing them with better individualized treatment and care. Additionally, our results may broaden current understanding of underlying pathological mechanisms for the NOAF in STEMI patients, thus implying new targets for the prevention of NOAF.

There are several limitations in our study. First, it is a single-center study based solely on Chinese individuals, thus limiting the generality of the present result. Second, due to the nature of retrospective analysis and limited samples, the result is inherently subject to selection bias and only provide an association between SIRI and NOAF instead of causality. Third, Several inflammatory indicators, such as, CRP, interleukins, were not assessed. Additionally, the level of SIRI was evaluated on admission for one-time and the fluctuation of SIRI had not been measured during hospitalization. Future large-scale prospective investigation is thus necessary to validate our result.

## Conclusion

In conclusion, SIRI is independently associated with NOAF in STEMI patients treated with PCI, with NOAF patients experiencing higher risks of in-hospital complications and long-term all-cause death as compared to those with SR.

## Data Availability

The raw data of this article will be made available by the corresponding author, without undue reservation.

## Abbreviations

NOAF: New-onset atrial fibrillation
STEMI: ST-elevated myocardial infarction
PCI: Percutaneous coronary intervention
SIRI: Systemic inflammatory response index
SR: Sinus rhythm
NLR: Neutrophil-lymphocyte ration
MLR: Lymphocyte-monocyte ration
DM: Diabetes mellitus
HDL-c: High-density lipoprotein cholesterol
LDL-c: Low-density lipoprotein cholesterol
CK: Creatinine, creatine kinase
BMI: Body mass index
MDRD: Modification of diet in renal disease
eGFR: Estimated glomerular filtration rate
LA: Left atrium
LVEF: Left ventricular ejection fraction
SYNTAX: Synergy between PCI with taxus and cardiac surgery
TIMI: Thrombolysis in myocardial infarction
ECG: Electrocardiogram
VT: Ventricular tachycardia
ROC: Receiver operating characteristic
AUC: Area under the curve
ACEI/ARB: Angiotensin-converting enzyme inhibitor/angiotensin receptor blocker
MRA: Mineralocorticoid receptor antagonist
AMI: Acute myocardial infarction
IL: Interleukin
TNF: Tumor necrosis factor
